# The Role of Intimate Dipole Carbonyl–Carbonyl and Hydrogen–Carbonyl Interactions in the Stereocomplexation and Crystallization: The Case of Poly(Cyclohexene Carbonate)

**DOI:** 10.1002/anie.202504418

**Published:** 2025-10-11

**Authors:** Massimo Christian D'Alterio, Miriam Scoti, Rocco Di Girolamo, Giovanni Talarico, Geoffrey W. Coates, Claudio De Rosa

**Affiliations:** ^1^ Dipartimento di Scienze Chimiche Università di Napoli Federico II Complesso Monte S. Angelo, Via Cintia Napoli I‐80126 Italy; ^2^ Department of Chemistry and Chemical Biology Baker Laboratory Cornell University Ithaca New York 14853‐1301 USA

**Keywords:** Crystal structure, Intimate dipole carbonyl and hydrogen–carbonyl interactions, Isotactic poly(cyclohexene carbonate), Stereocomplexation

## Abstract

Enantiopure isotactic poly(cyclohexene carbonate) (PCHC) has been synthesized with chiral Zn‐β‐diiminate catalyst. PCHC crystallizes both as enantiopure polymer (*R*)‐PCHC and (*S*)‐PCHC and upon stereocomplexation of the two enantiomers. We report the crystal structures of the enantiopure polymer and of the stereocomplex (*R*/*S*)‐PCHC and explain their crystallization based on the establishment of multiple attractive H‐‐‐O═C interactions between oxygen atoms of carbonyl groups and the hydrogen atoms of the cyclohexyl rings and C═O‐‐‐C═O intimate dipole interactions between carbonyl groups of chains of opposite chirality in the stereocomplex. The crystal structure of the enantiopure polymer is characterized by chains in 2/1 helical conformation packed in the orthorhombic unit cell with axes *a *= 11.55 Å*, b *= 9.42 Å, and *c *= 7.36 Å, according to the space group *P*2_1_2_1_2_1_, with steric interdigitation between chains of similar chirality favored by multiple attractive H‐‐‐O═C interactions. The stereocomplex crystallizes in an orthorhombic unit cell with axes *a* = 10.40 Å, *b *= 8.41 Å, and *c* = 7.36 Å, according to the space group *Pbc*2_1_, driven by establishment of additional C═O‐‐‐C═O dipole interactions between carbonyl groups of chains of opposite chirality, besides of the multiple attractive H‐‐‐O═C interactions.

## Graphical abstract

Isotactic poly(cyclohexene carbonate) crystalizes both as enantiopure polymer and upon stereocomplexation of the two opposite enantiomers. Both structures are characterized by multiple H‐‐‐O═C tight attractive interactions, whereas in the stereocomplex a tight interdigitation of the two enantiomeric chains occurs due to additional intimate dipole interactions between carbonyl groups of the opposite enantiomers.

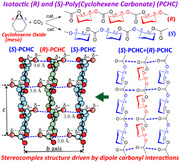

## Introduction

The synthesis of aliphatic polycarbonates through the alternating ring opening copolymerization (ROCOP) of epoxides and CO_2_ has been extensively investigated in the last decades.^[^ ^1^, ^2^, ^3^, ^4^, ^5^, ^6^, ^7^, ^8^, ^9^, ^10^, ^11^, ^12^ ^]^ The interest toward this reaction mainly resides in 1) the consumption of an “undesired” feedstock as CO_2_ in the perspective of mitigation of the global warming and 2) the properties of the obtained polycarbonates being potentially biodegradable and recyclable. In this perspective, the performance of the polycarbonates is strictly connected to the stereochemistry of the main chain, that can be tuned in polymerization thanks to stereoselective metal‐based catalysts. Among different possible epoxides, particularly intriguing is the achiral monomer *meso*‐cyclohexene oxide (CHO) that, upon ROCOP at the *S* or *R* stereocenter, can be de‐symmetrized employing a proper enantioselective catalyst giving the isotactic poly(cyclohexene carbonate) (PCHC). This isotactic polycarbonate is of growing interest because it is a semi‐crystalline polymer that exhibits enhanced thermal and mechanical properties relative to its atactic analogue^[^ ^7^ ^]^ and, in addition, is potentially chemically recyclable to the monomer.^[^ ^8^ ^]^

A further intriguing aspect of such CO_2_‐based polycarbonates is the possibility to induce stereocomplexation between macromolecules with opposite configurations. In particular, the first pair of stereocomplexable polycarbonates was the stereo‐gradient iso‐enriched poly(propylene carbonate) (PPC) synthesized by Nozaki et al.^[^ ^9^ ^]^ in 2011 employing a highly enantioselective cobalt–salen complex with ammonium substituents. Their claim was based on the fact that the obtained stereo‐gradient PPC as well as the stereo‐block PPC were found to possess higher thermal decomposition temperature than the typical PPCs.^[^ ^9^ ^]^

Further proof of stereocomplexable polycarbonates has been provided by Lu's group^[^ ^10^, ^11^, ^12^ ^]^ in 2012 who conducted pioneering research in the synthesis and stereocomplex crystallization of various polycarbonates. In particular, enantiopure isotactic (*S*)‐PCHC and (*R*)‐PCHC have been synthesized by enantioselective alternating copolymerization of CHO and CO_2_ using an enantiomerically pure cobalt(III) complex as catalyst. The formation of a crystalline stereocomplex (*R*/*S*)‐PCHC was demonstrated by X‐ray diffraction and analysis of the thermal behavior of the 1:1 blend of the two enantiomeric polymers characterized by very high stereoregularity with enantiomeric excesses (*ee*) of 98%.^[^ ^12^ ^]^

The crystallization of a polymer stereocomplex in a crystal form different from that of the corresponding enantiomers and melting at higher temperatures has been observed in the well‐known case of poly(lactide) (PLA).^[^ ^13^, ^14^, ^15^, ^16^ ^]^

In this context, it is worth mentioning that certain noncrystallizable or poorly crystallizable aliphatic polycarbonates can become highly crystallizable through stereocomplexation. This is the case, for instance, of poly(limonene carbonate) (PLC),^[^ ^17^, ^18^, ^19^ ^]^ synthesized via alternating copolymerization of CO_2_ with *R*‐ or *S*‐*trans*‐limonene oxide by Coates et al.^[^ ^17^ ^]^ Whereas the enantiopure polymers *R*‐PLC and *S*‐PLC do not crystallize, the equimolar blend of *R*‐PLC and *S*‐PLC crystallizes forming a stereocomplex thanks to the favorable interactions between the two opposite enantiomers.^[^ ^18^, ^19^ ^]^

Another series of crystalline stereocomplexed polycarbonates prepared by mixing enantiomeric isotactic (*R*)‐ and (*S*)‐polycarbonates of different structures was identified by Lu et al.^[^ ^20^, ^21^, ^22^ ^]^ These chiral polycarbonates were synthesized via enantioselective copolymerization of CO_2_ with various meso‐epoxides.^[^ ^20^, ^21^ ^]^ In some cases both the enantiopure polymers and the stereocomplex crystallize but the stereocomplexes exhibit higher melting temperatures (as in PLA), in other cases the enantiopure polymers do not crystallize but the stereocomplex does crystallize (as in PLC).^[^ ^20^, ^21^ ^]^

The complete structural understanding of the formation of stereocomplexes and the conditions that favor their crystallization remain an intriguing challenge. Solid‐state NMR experiments and density functional theory (DFT) calculations suggested that specific interlocking interactions induced by hydrogen bonds formed between carbonate units of the opposite enantiomers are the main driving force for the formation of stereocomplexes of these polycarbonates.^[^ ^20^, ^21^ ^]^ However, these calculations have revealed interlocking interactions between enantiomeric chains not included in a crystalline unit cell. In fact, despite several efforts in developing novel aliphatic polycarbonates, apart from the case of PLC, the crystal structures of these stereo‐complexes, as well as of the pure enantiomeric chiral polymers (when they crystallize), have not been resolved and, therefore, this driving force based on specific interactions has not been completely confirmed. On the other hand, the crystallization of *racemic compounds*, where chiral chains of opposite chirality are included in a unit cell pairwise giving optical compensation, or the crystallization of a *conglomerate*, where the same fractions of enantiomeric crystals may co‐exist, each made of only *R* macromolecules or only *S* macromolecules, giving intercrystallite optical compensation, have been observed in various chiral polymers and not necessarily related to the formation of hydrogen bonds or other specific interactions.^[^ ^23^, ^24^, ^25^ ^]^

In this paper we report the synthesis and the crystallization behavior of isotactic PCHC, and the complete resolution of the crystal structures of the pure enantiomers (*S*)‐PCHC (or (*R*)‐PCHC) and of the stereocomplex (*R*/*S*)‐PCHC, giving structural evidence of the role of specific interactions on the crystallization of both the enantiopure polymer and the stereocomplex. The case of PCHC represents an example of crystallization of the parent enantiopure polymers thanks to a favorable packing of chiral chains in a chiral crystal, with establishment of specific H‐‐‐O═C interactions between oxygen atoms of carbonyl groups and the hydrogen atoms of the cyclohexyl rings, and crystallization of the stereocomplex thanks to additional favorable interlocking dipole interactions between carbonyl groups of opposite enantiomers.

## Results and Discussion

### Copolymerization

**Figure 1:**
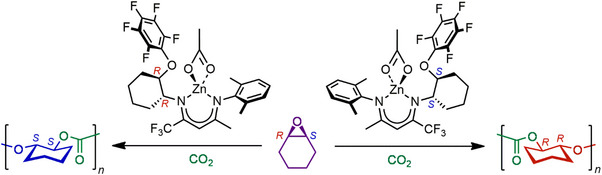
Alternating copolymerization of cyclohexene oxide and CO_2_ to form isotactic enantiopure (*R*)‐PCHC and (*S*)‐PCHC catalyzed by enantioselective *C*_1_ symmetric Zn β‐diiminate catalysts.

Samples of isotactic pure enantiomers (*R*)‐PCHC and (*S*)‐PCHC have been synthesized by copolymerization of *meso*‐CHO and CO_2_ as described in ref. [7] using the enantioselective *C*_1_ symmetric (*S*,*S*)‐Zn β‐diiminate and (*R*,*R*)‐Zn β‐diiminate (Zn‐BDI) catalysts, respectively, according to Figure 1 (see the Supporting Information for details). The samples were characterized by ^13^C and ^1^H‐NMR (Figures S1–S4) and gel‐permeation chromatography. The enantiomeric excess for both samples was determined on the trifluoroacetate‐derivatized diol obtained upon hydrolysis of the polymers.^[^ ^7^ ^]^ From these characterizations, the molecular masses, dispersities and enantiomeric excesses resulted: *M*_n_ = 33 kDa, *Ð* = 1.22 and *ee* = 91% for (*R*)‐PCHC, *M*_n_ = 44 kDa, *Ð* = 1.26, *ee* = 90% for (*S*)‐PCHC.^[^ ^7^ ^]^

### Structural and Thermal Characterization

**Figure 2:**
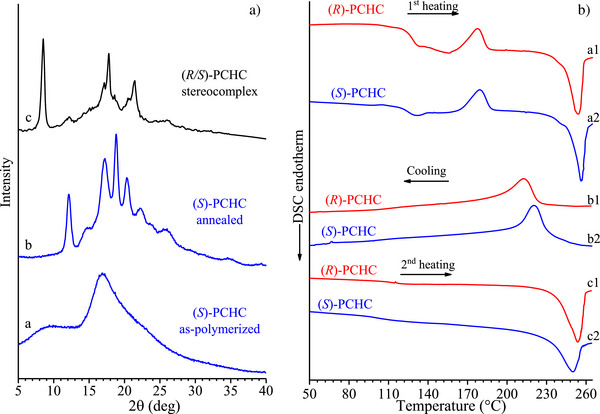
a) X‐ray powder diffraction profiles of the as‐polymerized sample of isotactic (*S*)‐PCHC (a), after annealing at 180 °C for 4 h (b), and of the stereocomplex (*R*/*S*)‐PCHC (c) reproduced from the literature,^[^ ^12^ ^]^ crystallized from solution of the 1:1 blend of the enantiopure polymers (*S*)‐PCHC and (*R*)‐PCHC after annealing at 180 °C for 1 h. b) DSC curves recorded at scanning rate of 10 °Cmin^−1^ during heating of the as‐polymerized samples (a1,a2), cooling from the melt (b1, b2) and successive heating of the melt‐crystallized samples (c1,c2) of (*R*)‐PCHC (a1,b1,c1) and (*S*)‐PCHC (a2,b2,c2).

The X‐ray diffraction profile of the as‐polymerized sample of (*S*)‐PCHC is reported in Figure 2a (profile a). The presence of two broad and diffuse haloes at 2*θ* ≈ 10° and 17° indicates that the sample is amorphous. Similar profile has been obtained for the enantiomeric sample (*R*)‐PCHC (Figure S5). Both enantiopure (*S*)‐PCHC and (*R*)‐PCHC crystallize after annealing at temperatures higher than 180 °C. The diffraction profiles of samples of (*S*)‐PCHC and (*R*)‐PCHC annealed at 180 °C for 4 h show four main sharp and well resolved reflections, centered at about 2*θ* ≈ 12.1°, 17.2°, 18.8°, and 20.3° (profile b of Figure 2a and Figure S6).

The DSC curves recorded during heating of the as‐polymerized samples of enantiopure polymers (*S*)‐PCHC and (*R*)‐PCHC, subsequent cooling from the melt and successive second heating are reported in Figure 2b. Both enantiopure polymers exhibit glass transition temperatures of nearly 125 °C, melt at nearly 255 °C, and crystallize by cooling from the melt at 215–220 °C (Figure 2b). Specifically, the amorphous as‐polymerized specimens show cold crystallization at 178 °C during heating and then melt at 255 °C. The occurrence of cold‐crystallization explains the diffraction data of Figure 2a and crystallization of both (*S*)‐PCHC and (*R*)‐PCHC upon annealing at 180 °C.

**Figure 3:**
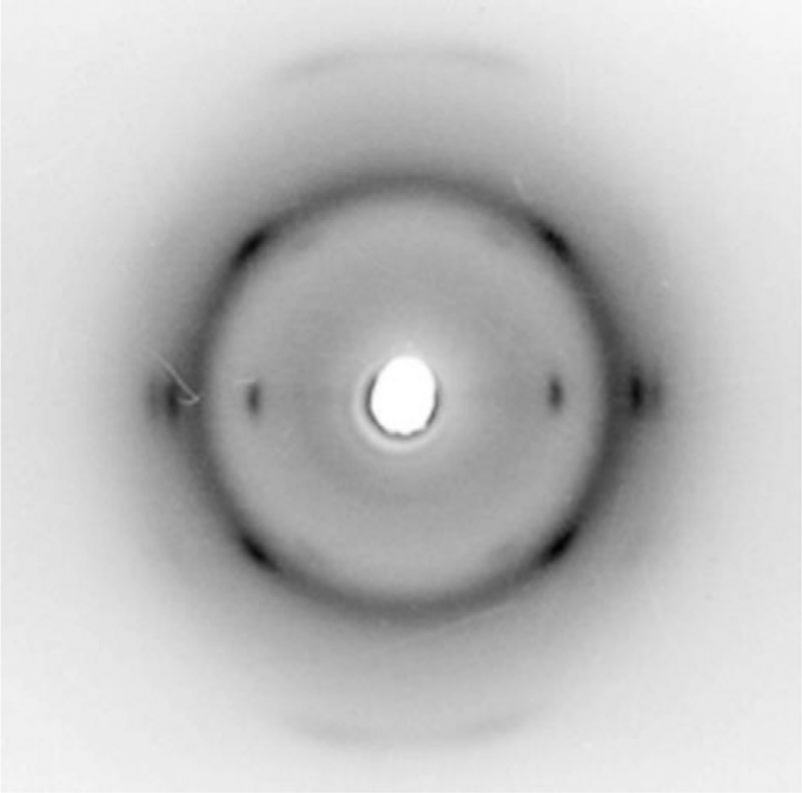
X‐ray fiber diffraction pattern of fibers of (*S*)‐PCHC prepared by stretching cast film of (*S*)‐PCHC at 180 °C at 500% deformation.

The X‐ray diffraction pattern of oriented fibers of (*S*)‐PCHC prepared by stretching at 180 °C films obtained by casting solution is reported in Figure 3. The pattern exhibits the same strong reflections observed in the X‐ray powder diffraction profile b of Figure 2a and indicates a chain periodicity of *c* = 7.4 Å. This suggests a regular chain conformation with repetition after two monomeric units.

A sample of 1:1 blend of (*R*)‐PCHC and (*S*)‐PCHC was prepared by dissolving equal amounts of the two enantiopure polymers in CH_2_Cl_2_ solution and successive casting at room temperature.^[^ ^12^ ^]^ After isothermal crystallization from the melt at 225 °C for 2 h, the blend crystallizes giving a diffraction profile (shown in Figure S12) similar to that of the stereocomplex reported by Lu et al. in the ref. [12], with intense diffraction peaks at 2*θ* = 8.7°, 20.9°, and 21.5°.

However, the diffraction profile of Figure S12 shows, besides the reflections of the stereocomplex at 2*θ* = 8.7°, 20.9°, and 21.5°, other unresolved weak reflections at 2*θ* = 15°, 17°, and 19°, probably corresponding to crystals of the enantiopure polymers (profile b of Figure 2a and Figure S6). This indicates that in our 1:1 blend the crystallization of the stereocomplex is not complete and part of the blend crystallizes giving small amounts of separate crystals of the enantiopure polymers (*S*)‐PCHC and (*R*)‐PCHC. This is probably due to the too high molecular weights and moderate tacticity of our samples. In fact, a (*R/S*)‐PCHC stereocomplex was obtained by Lu and Yan from the crystallization of the 1:1 blend of the two enantiomeric polymers characterized by very high stereoregularity (*ee* = 98%) and low molecular weight.^[^ ^12^ ^]^

For this reason, we have used the diffraction profile of the stereocomplex reported by Lu and Yan in ref. [12] to achieve information about the structure of the stereocomplex. This diffraction profile reproduced from ref. [12] is shown in Figure 2a (profile c). It is completely different from that of the enantiopure polymers (profile b of Figure 2a), showing intense diffraction peaks at 2*θ* = 8.5°, 17.1°, 17.8°, and 21.5°. This indicates that the two opposite enantiomers in the blend crystallize forming a stereocomplex (*R/S*)‐PCHC giving a different crystal structure where the two enantiomeric chains are included in the unit cell pairwise.

### Conformational Analysis

**Figure 4:**
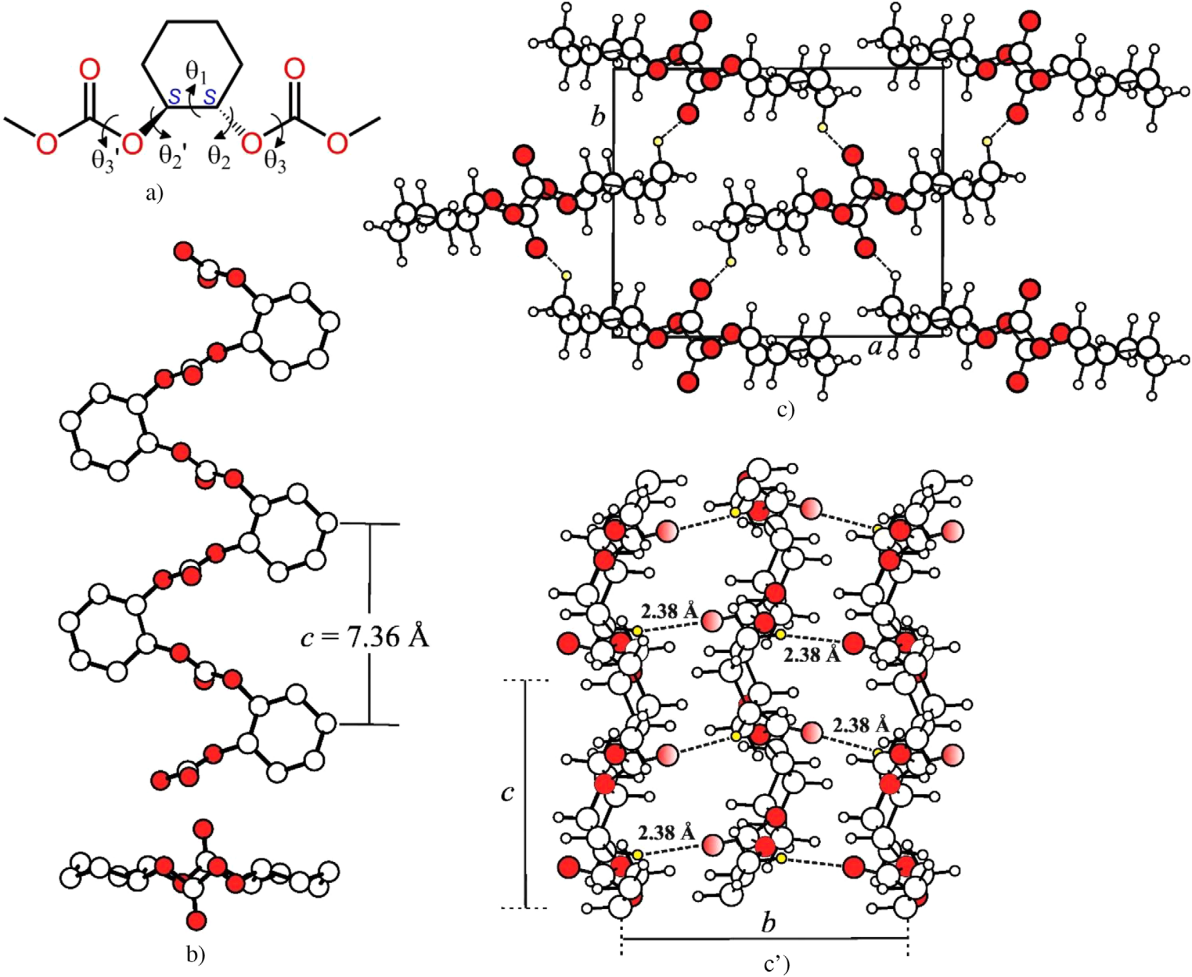
a) Portion of the chain of (*S*)‐PCHC and definition of the torsion angles θ_1_, θ_2_, θ_2_^’^ θ_3_, and θ_3_^’^. b) Model of the 2/1 helical conformation of (*S*)‐PCHC. Oxygen atoms are in red and hydrogen atoms are not shown. The value of the chain axis is indicated. c,c’) Model of the crystal structure of (*S*)‐PCHC viewed in *ab* (c) and *bc* (c’) projections. Chains in 2/1 helical conformation are packed in the orthorhombic unit cell with axes *a *= 11.55 Å*, b *= 9.42 Å, and *c *= 7.36 Å, according to the space group *P*2_1_2_1_2_1_. Oxygen atoms are in red. The dashed lines indicate the multiple H‐‐‐O═C attractive interactions at 2.38 Å.

Conformations of low energy of isotactic enantiopure (*R*)‐PCHC and (*S*)‐PCHC with periodicity of 7.4 Å have been obtained by calculation of the conformational energy using DFT.^[^ ^26^ ^]^ The conformational analysis has been performed on a portion of the chain of the *S*‐enantiomer shown in Figure 4a, whose conformation is defined by the five torsion angles θ_1_, θ_2_, θ_3_, θ_3_′, and θ_2_′.

Low‐energy conformations were built up by fixing the cyclohexyl rings in the chair conformation and assuming the torsion angle θ_1_ in the *gauche* conformation, *θ*_1_ ≈ 60° (G^+^). Moreover, the torsion angles θ_3_ and θ_3_′ were initially assumed at 180° (T).^[^ ^18^, ^19^, ^24^, ^25^, ^27^, ^28^ ^]^ The map of the conformational energy for (*S*)‐PCHC as a function of torsion angles θ_2_ and θ_2_′, with θ_1_, θ_3_ and θ_3_′ freely optimized is reported in the Figure S10. The map presents three nonequivalent energy minima (Table S1) but only the deepest energy minimum, which is located at θ_2_ = θ_2_′ = −150° (A^−^) for (*S*)‐PCHC (and θ_2_ = θ_2_′ = +150° (A^+^) for (*R*)‐PCHC), matches the experimental periodicity of 7.4 Å and a repetition after two monomers. This conformation of lowest energy corresponds to a two‐fold (2/1) helical conformation and a sequence of torsion angles (θ_3_′, θ_2_′, θ_1_, θ_2_, θ_3_)_n_ = (180°, ‐150°, 63°, ‐150°, 180°)_n_ = (TA^−^G^+^A^−^T)_n_ for (*S*)‐PCHC, and (θ_3_′, θ_2_′, θ_1_, θ_2_, θ_3_)_n_ = (180°, +150°, ‐63°, +150°, 180°)_n_ = (TA^+^G^−^A^+^T)_n_ for (*R*)‐PCHC.

This 2/1helical conformation has been then optimized by minimization of the energy while changing the five torsion angles and bond angles and lengths. The optimization gives a 2/1 helical conformation with a sequence of dihedral angles (θ_3_′, θ_2_′, θ_1_, θ_2_, θ_3_)_n_ = (167.6°, ‐94.7°, 68.0°, ‐141.2°, ‐176.4°)_n_ for (*S*)‐PCHC and the enantiomorphous conformation (θ_3_′, θ_2_′, θ_1_, θ_2_, θ_3_)_n_ = (‐167.6°, 94.7°, ‐68.0°, 141.2°, 176.4°)_n_ for the enantiomer (*R*)‐PCHC, and a periodicity of 7.36 Å, which perfectly matches the measured value of the *c* axis. A model of the 2/1 helical conformation for the (*S*)‐PCHC chain is shown in Figure 4b.

### Crystal Structure of the Enantiopure (*S*)‐PCHC or (*R*)‐PCHC

The reflections observed in the X‐ray powder diffraction profile b of Figure 2a of (*S*)‐PCHC can be interpreted by an orthorhombic unit cell with axes *a *= 11.55 Å*, b *= 9.42 Å, and *c *= 7.36 Å, which agrees with the density of 1.21 gcm^−3^ measured by floatation on a sample of (*S*)‐PCHC with a degree of crystallinity of nearly 50%.

**Figure 5:**
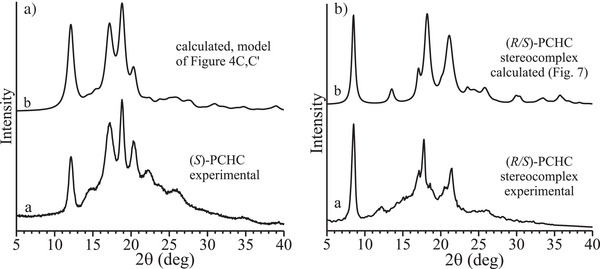
a) Comparison between the experimental X‐ray powder diffraction profile of (*S*)‐PCHC (a) and the diffraction profile calculated (b) for the packing model of Figure 4c,c'  of the crystal structure of the enantiopure (*S*)‐PCHC in the space group *P*2_1_2_1_2_1_. b) Comparison between the experimental X‐ray powder diffraction profile of the stereocomplex (*R*/*S*)‐PCHC (a)^[^ ^12^ ^]^ and the diffraction profile calculated (b) for the model of Figure 7 of the stereocomplex in the space group *Pbc*2_1_.

Various chiral models of packing of (*S*)‐PCHC in the orthorhombic unit cell have been considered. A possible model that agrees with the diffraction data is defined by the space group symmetry *P*2_1_2_1_2_1_ and is shown in Figure 4c and c’ in the *ab* and *bc* projections, respectively. The model has been optimized by comparison of the calculated X‐ray diffraction patterns with the experimental X‐ray powder diffraction profile b of Figure 2a and the X‐ray fiber diffraction pattern of Figure 3. The comparison between the experimental and calculated X‐ray powder diffraction profiles is shown in Figure 5a, whereas the comparison between the experimental and calculated bidimensional X‐ray fiber diffraction patterns is shown in Figure S11. The achieved good agreement indicates that the chiral model of Figure 4c,c’ for the space group *P*2_1_2_1_2_1_ is a good description of the crystal structure of the enantiopure (*S*)‐PCHC or (*R*)‐PCHC. The fractional coordinates of the asymmetric unit in the model of the crystal structure of (*S*)‐PCHC of Figure 4c,c’ are reported in the Table S2.

An intriguing feature of the model of Figure 4c,c’ is that chains of similar chirality are tightly packed along the *b* axis with establishment of multiple H‐‐‐O═C attractive interactions at short distance of 2.38 Å between oxygen atoms of carbonyl groups and the hydrogen atoms on the methylene groups of the cyclohexyl rings, as highlighted in the *bc* projection of Figure 4c’. This could explain the crystallization of the enantiopure polymers (*S*)‐PCHC or (*R*)‐PCHC, contrary to other polycarbonates, as the enantiopure PLC that is amorphous and inherently non‐crystallizable and crystallizes only upon stereocomplexation.

**Figure 6:**
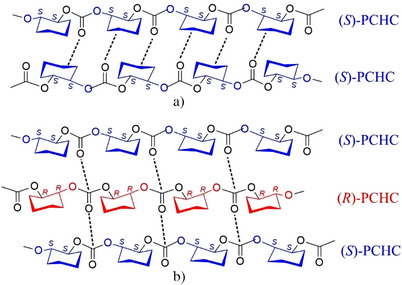
Schematics of the multiple H‐‐‐O═C tight attractive interactions at short distance of 2.38 Å between oxygen atoms of carbonyl groups and the hydrogen atoms of the methylene groups of the cyclohexyl rings of chains of PCHC of the same chirality in the crystal structure of the enantiopure (*S*)‐PCHC of Figure 4c,c’ (A) and of the additional intimate C═O‐‐‐C═O dipole interactions between carbonyl groups of chains of opposite chirality in the structure of the stereocomplex (*R*/*S*)‐PCHC of Figure 7.

The crystallization of the chiral enantiopure isotactic PCHC is, therefore, driven by favorable steric interdigitation between chains of similar chirality inside the *ac* layers along *a* and between chains belonging to adjacent *ac* layers along *b*, the latter favored by multiple H‐‐‐O═C tight attractive interactions at short distance of 2.38 Å, as shown in the scheme of Figure 6a.

### Crystal Structure of the Stereocomplex (*R*/*S*)‐PCHC

The reflections at 2*θ* = 8.5°, 17.1°, 17.8°, and 21.5° observed in the diffraction profile c of Figure 2a of the stereocomplex (*R*/*S*)‐PCHC can be again interpreted by an orthorhombic unit cell with axes *a* = 10.40 Å, *b *= 8.41 Å, and *c* = 7.36 Å, similar to that of the enantiopure polymers.

**Figure 7:**
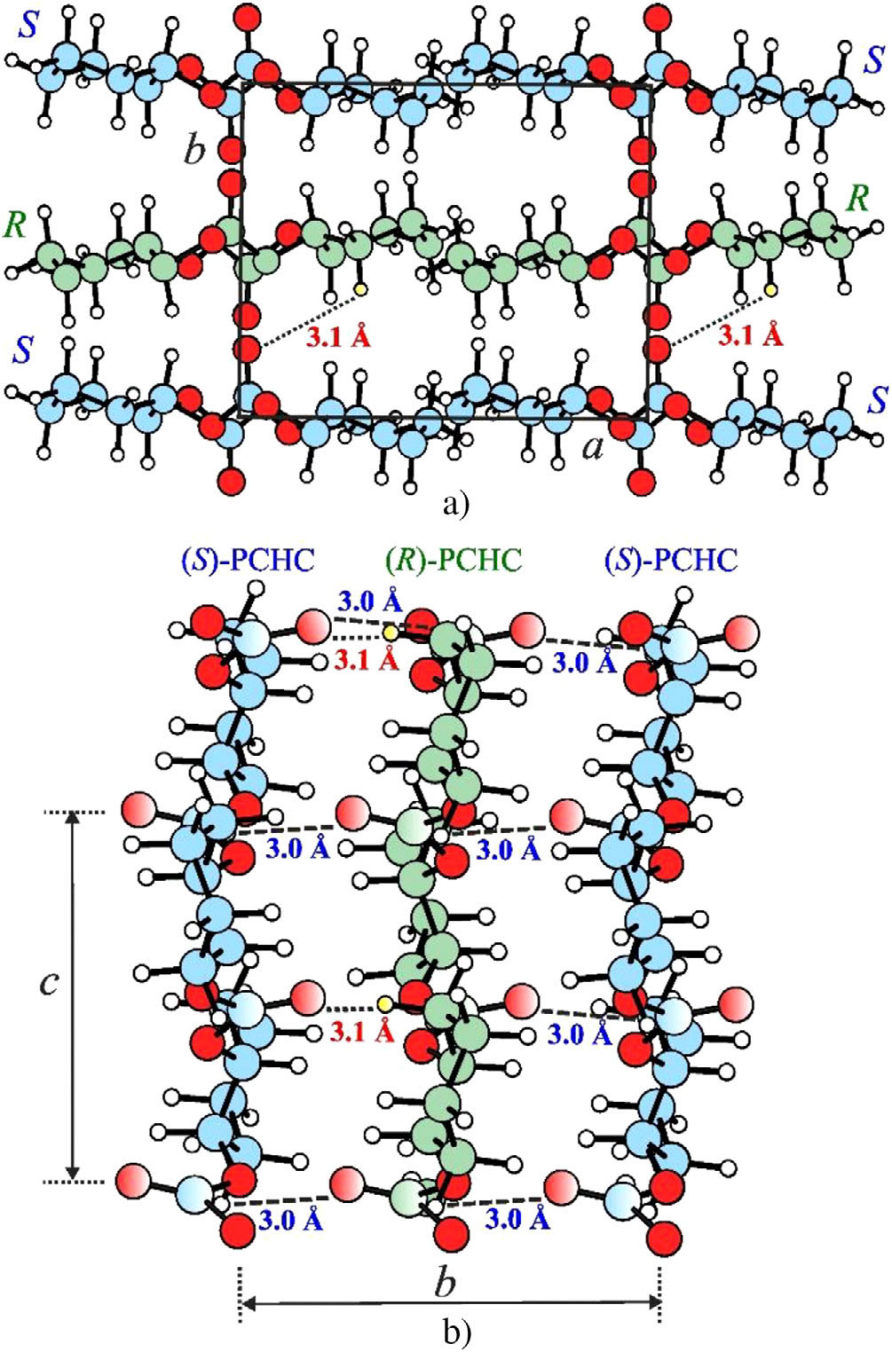
Model of the crystal structure of the stereocomplex (*R*/*S*)‐PCHC viewed in *ab* a) and *bc* b) projections. Enantiomorphic chains in 2/1 helical conformation are packed in the orthorhombic unit cell with axes *a* = 10.40 Å, *b* = 8.41 Å, and *c* = 7.36 Å, according to the space group *Pbc*2_1_. In green (*R*)‐chains, in blue (*S*)‐chains, oxygen atoms are red and hydrogen atoms are white. The dashed lines indicate the C═O‐‐‐C═O intimate dipole interactions between carbonyl groups of chains of opposite chirality at 3.0 Å and the dotted lines indicate the H‐‐‐O═C interactions at 3.1 Å between oxygen atoms of carbonyl groups and the hydrogen atoms on the methylene groups of the cyclohexyl rings belonging to enantiomeric chains.

A possible achiral model of the crystal structure of the stereocomplex (*R*/*S*)‐PCHC that accounts for the diffraction data is shown in Figure 7 in the *ac* and *bc* projections. Two enantiomeric chains of (*R*)‐PCHC and (*S*)‐PCHC in the 2/1 helical conformation are hosted in the orthorhombic unit cell pairwise and are packed according to the space group symmetry *Pbc*2_1_. The structure of the stereocomplex is characterized by *ac* layers of chains of similar chirality stacked along the *b* axis to *ac* layers of chains of opposite chirality, giving alteration of enantiomeric chains along the *b* axis direction. The *ab* projection of the crystal structure of Figure 7a clearly show the presence of H‐‐‐O═C interactions between oxygen atoms of carbonyl groups and the hydrogen atoms on the methylene groups of the cyclohexyl rings belonging to enantiomeric chains at 3.1 Å, similar or only slightly longer than those present in the structure of the enantiopure polymer (*S*)‐PCHC of Figure 4c. The projection along the *c* axis of Figure 7b clarifies the close packing along the *b* axis of chains of opposite chirality, and highlights the establishment of additional C═O‐‐‐C═O intimate dipole interactions between carbonyl groups of chains of opposite chirality facing along the *b* axis at distance of 3.0 Å (as in the scheme of Figure 6b). Therefore, in the structure of the stereocomplex of Figure 7 the closest distance H‐‐‐O═C between oxygen and hydrogen atoms is similar to the closest distance C═O‐‐‐C═O between carbonyl groups of chains of opposite chirality. This indicates that the crystallization of the stereocomplex is driven by establishment of attractive H‐‐‐O═C interactions between hydrogen atoms and carbonyl groups belonging to enantiomeric chains and additional C═O‐‐‐C═O intimate dipole interactions between carbonyl groups of chains of opposite chirality.

The specific intimate C═O‐‐‐C═O dipole interactions are absent in the crystal structure of the enantiopure polymer of Figure 4c,c’, which is, instead, mainly defined by the multiple H‐‐‐O═C attractive interactions. The different multiple H‐‐‐O═C attractive interactions that drive the crystallization of the enantiopure PCHC and the additional C═O‐‐‐C═O intimate dipole interactions present in the structure of the stereocomplex are compared in the scheme of Figure 6.

A deep analysis of the non‐covalent interactions (NCI) present in the structures of the enantiopure (*S*)‐PCHC of Figure 4c,c’ and of the stereocomplex (*R*/*S*)‐PCHC of Figure 7 has confirmed the scheme of Figure 6 (see Supporting Information for details). We have analyzed the chain‐chain interactions in the two models of Figures 4c,c’ and 7 by selecting representative pairs of polymeric chains to preserve, as accurately as possible, the intermolecular interactions occurring in the crystals, as dictated by the crystal symmetry. Gradient iso‐surfaces in the crystal structures of the enantiopure (*S*)‐PCHC and of the stereocomplex (*R*/*S*)‐PCHC showing non‐covalent interactions are reported in Figure S14. Significant attractive interactions are clearly present in both the structures of the enantiopure (*S*)‐PCHC (Figure S14A) and of the stereocomplex (*R*/*S*)‐PCHC (Figure S14B). In particular, in the enantiopure (*S*)‐PCHC interactions between the C═O and H─C groups are evident (Figure S14A), whereas additional attractive interactions appear between two carbonyl C═O groups in the structure of the stereocomplex (Figure S14B). This confirms the presence of H‐‐‐O═C attractive interactions in both structures of the enantiopure (*S*)‐PCHC and of the stereocomplex, and additional C═O‐‐‐C═O intimate dipole interactions in the structure of the stereocomplex (*R*/*S*)‐PCHC.

We have obtained further experimental evidence of the presence of additional intimate C═O‐‐‐C═O dipole interactions in the structure of the stereocomplex, besides the common H‐‐‐O═C attractive interactions, from the FTIR spectra of the enantiopure polymer and of the stereocomplex. The FTIR spectra of samples of the enantiopure (*S*)‐PCHC and of the 1:1 blend of the two enantiomeric polymers melt‐crystallized in the stereocomplex (*R*/*S*)‐PCHC are compared in Figure S13. It is apparent that the band of the carbonate group at 1749 cm^−1^ in the enantiopure (*S*)‐PCHC moves to 1759 cm^−1^ in the spectrum of the stereo‐complex with a wavenumber shift of 10 cm^−1^. This shift to higher wavenumbers indicates a strengthening of the C═O double bond as a result of establishment of strong non‐covalent interactions involving the carbonate groups. Since the structure of the enantiopure (*S*)‐PCHC is already characterized by the presence of H‐‐‐O═C attractive interactions at short distance of 2.38 Å between oxygen and hydrogen atoms (Figure 4c,c’), the shift of the carbonyl band in the stereocomplex indicates the presence, besides the H‐‐‐O═C attractive interactions, of additional interactions, such as the C═O‐‐‐C═O intimate dipole interactions between carbonyl groups of chains of opposite chirality (as shown in the model of the crystal structure of Figure 7). We hypothesize that this directional inductive bonding strengthens the double bond and suppresses the conjugation effect.

DFT calculations of the packing energies of the models of packing of the enantiopure (*S*)‐PCHC of Figure 4c,c’ and of the stereocomplex (*R*/*S*)‐PCHC of Figure 7 have indicated that the two structures have low and similar lattice energies, the packing energy of the stereocomplex being only 1 kcal/(mol of chains) higher. These calculations have also demonstrated that the low lattice energy of the stereocomplex is favored by the intimate dipole interactions between the carbonyl groups that resulted characterized by Mulliken charges of + 0.75 onto the carbon atom and ‐0.45 on the oxygen atom and a typical interaction distance O‐‐‐C of 3.0 Å.

A comparison between the experimental X‐ray powder diffraction profile of the stereo‐complex (*R*/*S*)‐PCHC of Figure 2a (profile c),^[^ ^12^ ^]^ and the X‐ray powder diffraction profile calculated for the model of Figure 7 in the space group *Pbc*2_1_ is shown in Figure 5b. A good agreement has been clearly achieved, indicating that the nonchiral model of Figure 7 is a good description of the crystal structure of the stereocomplex (*R*/*S*)‐PCHC.

## Conclusions

We have described the synthesis and crystallization behavior of isotactic PCHC. This polycarbonate crystallizes both as enantiopure polymers (*R*)‐PCHC and (*S*)‐PCHC, and upon stereocomplexation of the two enantiomers. We report the crystal structures of the enantiopure polymers (*R*)‐PCHC and (*S*)‐PCHC and of the stereocomplex (*R*/*S*)‐PCHC giving a structural explanation of stereocomplexation.

The crystal structure of the enantiopure (*R*)‐PCHC and (*S*)‐PCHC is characterized by chains in 2/1 helical conformation packed in the orthorhombic unit cell with axes *a *= 11.55 Å*, b *= 9.42 Å, and *c *= 7.36 Å, according to the space group *P*2_1_2_1_2_1_. The crystallization of the chiral enantiopure PCHC is driven by establishment of steric interdigitation between chains of similar chirality favored by multiple H‐‐‐O═C tight attractive interactions at short distance of 2.38 Å along the *b* axis between oxygen atoms of carbonyl groups and the hydrogen atoms of the methylene groups of the cyclohexyl rings (Figure 6a).

The crystal structure of the stereocomplex (*R*/*S*)‐PCHC is characterized by the packing of chains of opposite chirality (*R*)‐PCHC and (*S*)‐PCHC in 2/1 helical conformation in a similar orthorhombic unit cell with axes *a* = 10.40 Å, *b *= 8.41 Å, and *c* = 7.36 Å, according to the space group *Pbc*2_1_. The close packing of chains of opposite chirality is established by attractive H‐‐‐O═C interactions between hydrogen atoms and carbonyl groups along *a* axis, as in the case of the enantiopure polymer, and by additional C═O‐‐‐C═O intimate dipole interactions between carbonyl groups of chains of opposite chirality facing along the *b* axis (Figure 6b).

Therefore, the crystallization of the stereocomplex is driven by the tight interdigitation of the enantiomeric chains favored by establishment of attractive H‐‐‐O═C interactions between hydrogen atoms and carbonyl groups belonging to enantiomeric chains and additional C═O‐‐‐C═O intimate dipole interactions between carbonyl groups of chains of opposite chirality. Such additional dipole interactions have been confirmed by FTIR spectra that have revealed strengthening of the C═O bonds in the stereocomplex.

Such intimate interactions between carbonyl groups remind the well‐known interaction extensively described in proteins, where amide carbonyl groups can engage in C═O‐‐‐C═O interactions.^[^ ^29^, ^30^ ^]^ The involvement of charges suggest that these interactions are primarily dipolar. However, analysis of the crystal structures of many proteins have animated the discussion about a possible role of the *n*‐π* interactions between carbonyl group, which involves significant delocalization of an electron lone pair (*n*) of the oxygen of the donor carbonyl group into the antibonding orbital (π*) of the acceptor carbonyl group.^[^ ^29^, ^30^, ^31^, ^32^, ^33^, ^34^, ^35^, ^36^, ^37^, ^38^ ^]^ Evidence for *n*→π* interactions has been detected in small molecules,^[^ ^31^, ^32^ ^]^ peptides,^[^ ^33^, ^34^ ^]^ peptoids,^[^ ^35^ ^]^ proteins,^[^ ^29^, ^30^, ^36^ ^]^ and nucleic acids,^[^ ^37^ ^]^ and they have been postulated to stabilize transition states.^[^ ^38^ ^]^ These interactions have as first signature a short contact distance between the donor atom and the acceptor carbonyl carbon allowing for orbital overlap,^[^ ^30^ ^]^ as actually occurs in the crystal structure of the stereocomplex (*R*/*S*)‐PCHC.

## Supporting Information

Experimental details of the synthesis of (*R*)‐PCHC and (*S*)‐PCHC. Details of the molecular, structural, and morphological characterization by NMR, GPC, X‐ray diffraction, DSC, FTIR, and optical microscopy. Details of calculation of the conformational energy and of the method for resolution of the crystal structure. Additional data of X‐ray powder diffraction of (*R*)‐PCHC and (*S*)‐PCHC and X‐ray fiber diffraction pattern of stretched fibers of (*S*)‐PCHC. Maps of the conformational energy. Comparison between experimental and calculated X‐ray fiber diffraction patterns for the crystal structure of the enantiopure (*S*)‐PCHC. Fractional coordinates of the model of crystal structures of (*S*)‐PCHC. X‐ray powder diffraction profile of the stereocomplex. FTIR spectra of the samples of the enantiopure (*S*)‐PCHC and of the stereocomplex (*R*/*S*)‐PCHC. Analysis of the non‐covalent interactions in the models of the crystal structures of enantiopure (*S*)‐PCHC and of the stereocomplex (*R*/*S*)‐PCHC. The authors have cited additional references within the Supporting Information.^[^ ^39^, ^40^, ^41^, ^42^, ^43^, ^44^, ^45^, ^46^, ^47^, ^48^, ^49^, ^50^, ^51^ ^]^

## Conflict of Interests

The authors declare no conflict of interest.

## Supporting information



Supporting Information

## Data Availability

The data that support the findings of this study are available from the corresponding author upon reasonable request.
