# Supplementary material for: The Role of Intimate Dipole Carbonyl–Carbonyl and Hydrogen–Carbonyl Interactions in the Stereocomplexation and Crystallization: The Case of Poly(Cyclohexene Carbonate)
Source: Angew Chem Int Ed Engl. 2025 Oct 11;64(49):e202504418. doi: 10.1002/anie.202504418 (PMC12668303; doi:10.1002/anie.202504418)
Supplement: Supplementary file 1 — Supporting Information [file ANIE-64-e202504418-s001.pdf]

Supporting Information

The Role of Intimate Dipole Carbonyl-Carbonyl and Hydrogen-Carbonyl Interactions in the Stereocomplexation and Crystallization: The Case of Poly(Cyclohexene Carbonate).

Massimo Christian D'Alterio,[a] Miriam Scoti,[a] Rocco Di Girolamo,[a] Giovanni Talarico,[a] Geoffrey W. Coates,[b] and Claudio De Rosa*,[a]

[a] Dr. M. C. D'Alterio, Dr. Miriam Scoti, Prof. R. Di Girolamo, Prof. G. Talarico, Prof. Claudio De Rosa
Dipartimento di Scienze Chimiche, Università di Napoli Federico II, Complesso Monte S. Angelo, Via Cintia, I-80126 Napoli, Italy
E-mail: claudio.derosa@unina.it

[b] Prof. G. W. Coates
Department of Chemistry and Chemical Biology, Baker Laboratory, Cornell University, Ithaca, New York, 14853-1301, USA

Supporting information for this article is given via a link at the end of the document.

Abstract: Enantiopure isotactic poly(cyclohexene carbonate) (PCHC) has been synthesized with chiral Zn-β-diiminate catalyst. PCHC crystallizes both as enantiopure polymer (*R*)-PCHC and (*S*)-PCHC and upon stereocomplexation of the two enantiomers. We report the crystal structures of the enantiopure polymer and of the stereocomplex (*R*/*S*)-PCHC and explain their crystallization based on the establishment of multiple attractive H---O=C interactions between oxygen atoms of carbonyl groups and the hydrogen atoms of the cyclohexyl rings and C=O---C=O intimate dipole interactions between carbonyl groups of chains of opposite chirality in the stereocomplex. The crystal structure of the enantiopure polymer is characterized by chains in 2/1 helical conformation packed in the orthorhombic unit cell with axes *a* = 11.55 Å, *b* = 9.42 Å and *c* = 7.36 Å, according to the space group $P2_12_12_1$, with steric interdigitation between chains of similar chirality favored by multiple attractive H---O=C interactions. The stereocomplex crystallizes in a orthorhombic unit cell with axes *a* = 10.40 Å, *b* = 8.41 Å and *c* = 7.30 Å, according to the space group $Pbc2_1$, driven by establishment of additional C=O---C=O dipole interactions between carbonyl groups of chains of opposite chirality, besides of the multiple attractive H---O=C interactions.

1. Experimental Procedures

1.1 Polymerization.

Samples of enantiopure isotactic (*R*)- and (*S*)-poly(cyclohexene carbonate)s (PCHC) were synthesized by copolymerization of *meso*-cyclohexene oxide (CHO) and CO_2 as described in ref. S1 using the enantioselective C_1 symmetric (*S,S*)-Zn β-diiminate and (*R,R*)-Zn β-diiminate (Zn-BDI) catalysts, respectively, according to Fig. 1. An oven dried Fisher-Porter bottle was taken into the glove box. It was charged with a stir bar, 1.0 mL toluene, 1.0 mL of *meso*-CHO, and 0.25 mol % of (*R,R*)-Zn-BDI or (*S,S*)-Zn-BDI catalyst. The vessel was sealed with the reactor head and the apparatus was removed from the box. The vessel was placed in a 0 °C water bath and allowed to equilibrate for 15 minutes. The vessel was then charged to 8 atm CO_2, vented to ~2.4 atm, and recharged to 8 atm. The vessel was left open to 8 atm for 1 minute to allow saturation of the reaction mixture. The CO_2 pressure was then monitored as a gauge of conversion. The reaction was terminated when the mixture became too viscous for stirring, with a viscosity approaching that of honey. The vessel was vented to atmospheric pressure. The reaction mixture was diluted with 4 mL of toluene, and then precipitated into 200 mL rapidly stirring methanol. The resulting white precipitate was isolated by vacuum filtration, dried on the filter pad for 15 minutes, and then dried at 45 °C for 5 hours and then at 22 °C overnight.[S1]

1.2. Molecular characterization

The samples were characterized by ^{13}C and ^{1}H-NMR and gel-permeation chromatography (GPC) in THF at 30 °C. NMR spectra were recorded in $CDCl_3$ at room temperature using a Bruker Advance 400 MHz Ascend WB spectrometer. Chemical shifts (δ) are expressed as parts per million. The spectra are referenced using the residual solvent peak at δ = 7.26 for ^{1}H NMR and δ = 77.22 for ^{13}C NMR.

The enantiomeric excess (*ee*) for both samples was determined on the trifluoroacetate-derivatized diol obtained upon hydrolysis of the polymers.[S1]

From these characterizations, the molecular masses, dispersities and enantiomeric excesses of the synthesized samples resulted: M_n = 33 kDa, $Đ$ = 1.22 and *ee* = 91% for (*R*)-PCHC, M_n = 44 kDa, $Đ$ = 1.26, *ee* = 90% for (*S*)-PCHC.[S1]

1.3. Structural characterization by X-ray diffraction and DSC

The as-polymerized samples of both enantiopure (*S*)-PCHC and (*R*)-PCHC were amorphous at the X-ray diffraction analysis. Crystalline samples were obtained after annealing at 180 °C. Films of the (*S*)-PCHC and (*R*)-PCHC were prepared by slow casting at room temperature from a CH_2Cl_2 solution of 1 mg/mL concentration.

Melting and crystallization temperatures and glass transition temperature were estimated by differential scanning calorimetry (DSC-822 calorimeter by Mettler Toledo) performing scans in a flowing N_2 atmosphere at 10 °C/min scanning rate. Both enantiopure polymers exhibit a glass transition

temperature of nearly 125 °C, melting temperature of 255 °C and a crystallization temperature of 215-220 °C.

Oriented fibers of (*S*)-PCHC were prepared by stretching the unoriented cast film on a hot-bench Kofler system at 180 °C, above the glass transition temperature.

X-ray powder diffraction profiles were obtained with Ni filtered Cu Kα radiation (λ = 1.5418 Å) with an Empyrean diffractometer by Malvern Panalytical, operating in the reflection geometry with continuous scans of the 2θ angle and scanning rate of 0.02 degree/s. The degree of crystallinity (x_c) was determined from the diffraction profiles by the ratio between the crystalline diffraction area (A_c) and the area of the whole diffraction profiles ($A_t = A_c + A_{am}$), where A_{am} is the amorphous scattering area, $x_c = (A_c/A_t)100$. The area of the crystalline phase A_c was evaluated by subtracting a background baseline and the scattering halo of the amorphous phase (A_{am}) from the whole diffraction profile. The diffraction profile of the amorphous phase was obtained from the diffraction profile of the amorphous cast film.

The two-dimensional X-ray fiber diffraction pattern of oriented fibers was recorded on a BAS-MS imaging plate (FUJIFILM) with Ni-filtered Cu-Kα radiation using a cylindrical camera (radius 57.3 mm) and processed with a digital imaging reader Perkin Elmer Cyclone Plus (storage phosphor system).

1.4 Polarized optical microscopy.

Polarized optical microscopy (POM) images were recorded at room temperature in polarized light using a Zeiss Axioscop40 microscope provided with a Linkam hot stage. Thin films (20-50 μm thick) of the samples were prepared for POM experiments. Small amounts of the powder samples were sandwiched between glass coverslips, heated at heating rate of 40 °C/min at temperature of 265 °C to melt the samples and then cooled to room temperature at 10 °C/min.

1.5 Fourier-transform infrared spectroscopy.

Fourier-transform infrared (FTIR) spectra were recorded in transmittance mode using a Jasco FT/IR-4700 type A spectrometer equipped with a TGS detector. Samples were prepared by mixing the powdered samples with dry KBr. Spectra were acquired by accumulating 16 scans at a resolution of 4 cm^{-1}, in the range 4000-400 cm^{-1}.

2. Computational Methods.

Calculations of the conformational energy have been performed by using density functional theory (DFT) with the Gaussian 09, Revision E.01 set of programs.[S2] The generalized gradient approximation functional PBE[S3,S4] have been adopted and opportunely corrected with the Grimme dispersion term.[S5,S6] All the atoms (C, H, O) have been electronically described with the 6-31G(d) basis set by Petersson and coworkers, defined as part of various Complete Basis Set methods.[S7,S8] Optimization of the final chain conformation has been performed with Periodic Boundary Condition by adding a translational vector parallel to the chain axis.

The packing energy has been calculated with DFT-based methods by using DMol3 software package as implemented in and Accelrys BIOVIA Materials Studio (MS)[S9,S10] software package using GGA-PBE functional DND 4.4 as basis set and Grimme method to account for the dispersion correction.[S5,S6]

Structural modeling, X-ray powder diffraction simulations and full-profile refinement have been performed by using Material Study (MS) software package. The crystal structure was refined by Rietveld powder diffraction refinement by using the Rietveld program implemented in the Powder Refinement tool of MS package. The program is based on standard non-linear least squares algorithms employed in Rietveld refinement.[S11,S12] In the refinement the sizes of the crystallite have been set at 150 Å along *a* and *b* axes and 100 Å along the *c* axis of the unit cell. These values correspond to a coherence length along *a*, *b*, and *c* and are not a true crystallite size. The global anisotropic temperature factors have been set at $B_x = B_y = 8$ Å^2 along the *a* and *b* axes and $B_z = 20$ Å^2 along the *c* axis. The experimental background, including the contribution of the amorphous phase, has been fitted with a polynomial function of order 20 and the background coefficients have been refined.

For the calculation of X-ray powder diffraction profiles, profile functions having half-height width regulated by the average crystallite size along *a*, *b*, and *c* axes, $L_a = L_b = 150$ Å and $L_c = 100$ Å, respectively have been used, as in the refinement procedure.

Simulated X-ray fiber diffraction pattern has been obtained with home-made programs and using crystallite sizes along *a*, *b* and *c* axes of $L_a = L_b = 150$ Å, and $L_c = 100$ Å. These values correspond to a coherence length along *a*, *b*, and *c* and are not a true crystallite size.

The comparison between X-ray diffraction intensities and calculated structure factors has been performed by taking the experimental intensities from the X-ray fiber diffraction pattern, as well as, for a better quantitative comparison, from the X-ray powder diffraction profiles. In the latter case, the observed structure factors (F_o) have been obtained as the square root of the experimental intensities corrected by the Lorentz-Polarization factor for powder diffraction $LP = (1+\cos^2 2\theta)/(\sin^2\theta \cos\theta)$, $F_o = (I/LP)^{1/2}$. The experimental intensities have been evaluated measuring the area of the peaks in the

X-ray powder diffraction profiles, after subtraction of the amorphous halo. Calculated structure factors (F_c) have been obtained as $F_c = (\sum|F_c(hkl)_i|^2 \times M_i)^{1/2}$ where M_i is the multiplicity factor for powder diffraction, and the summation is taken over all the reflections included in the 2θ range of the corresponding observed reflection peak. In the case of the fiber diffraction pattern, the observed intensities are compared with the square modulus of the structure factors $F_c^2 = \Sigma|F_c(hkl)_i|^2 \times M_i$, where M_i is the multiplicity factor for fiber diffraction. Anisotropic thermal factors along ξ and ζ directions, B_ξ = 8 Å^2 and B_ζ = 20 Å^2 respectively, have been assumed. In all calculations of structure factors, values of the atomic scattering factors as given in Ref. [S13] have been assumed.

3. Supplementary Experimental Data

3.1. ¹H-NMR and ¹³C-NMR spectra

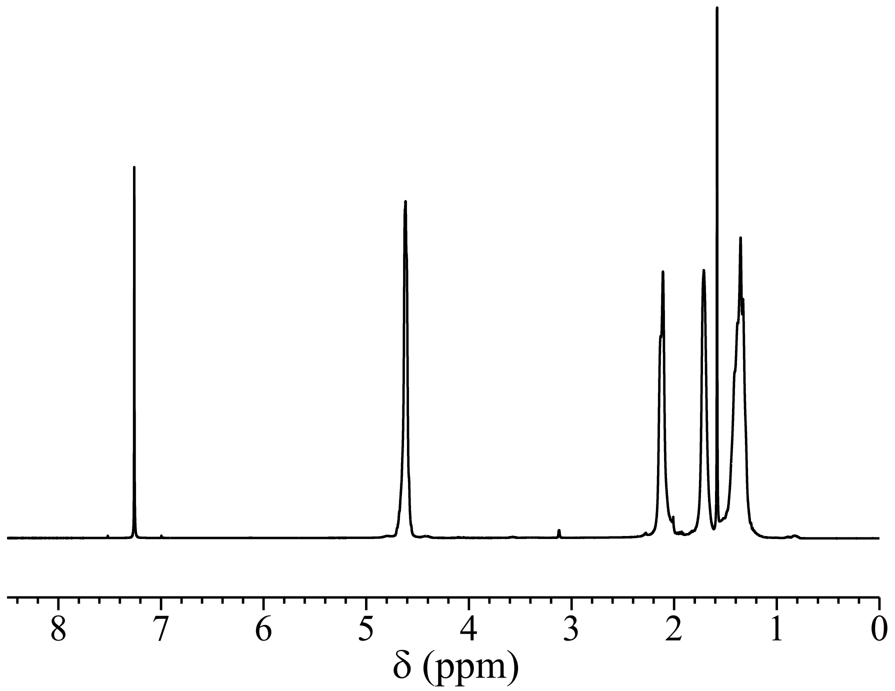

Figure S1. ^{1}H NMR spectrum of (*R*)-PCHC (solvent: δ = 7.26 ppm).

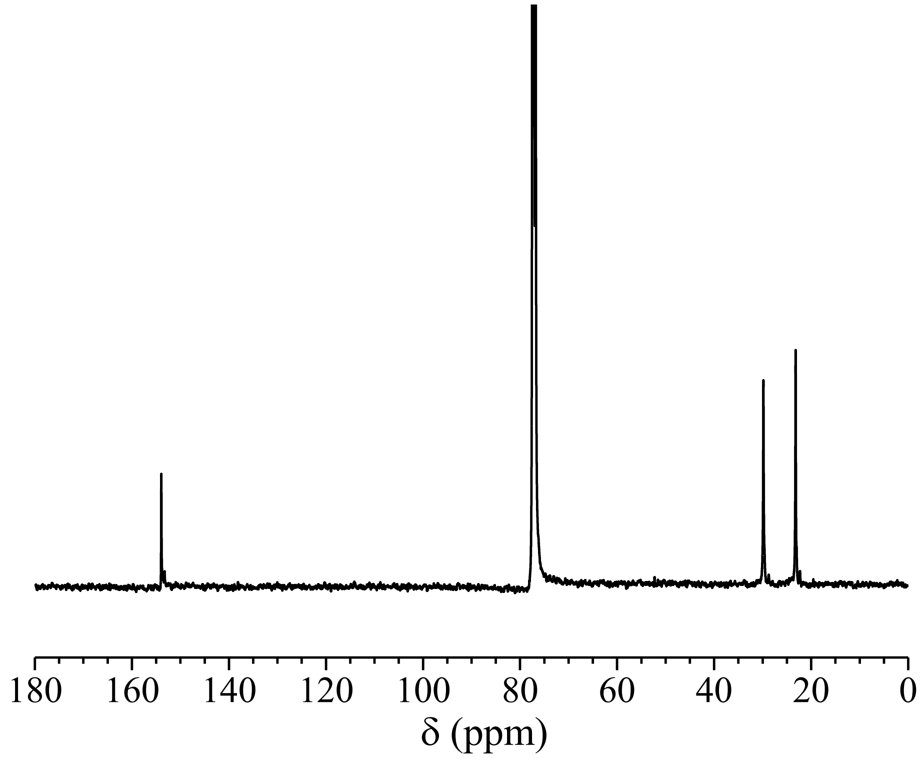

Figure S2. ^{13}C NMR spectrum of (*R*)-PCHC (solvent: δ = 77.22 ppm).

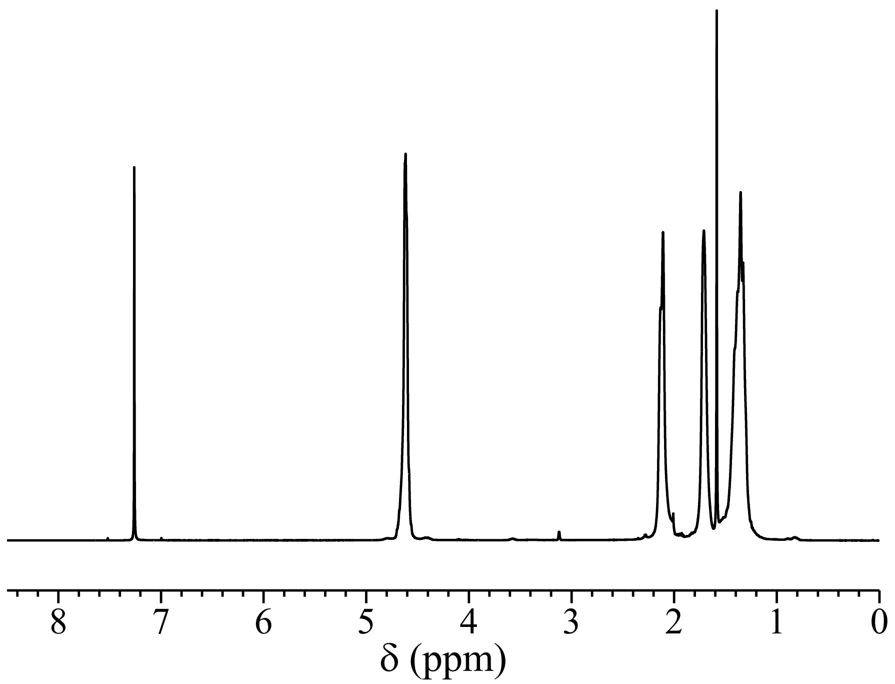

Figure S3. ^{1}H NMR spectrum of (*S*)-PCHC (solvent: δ = 7.26 ppm).

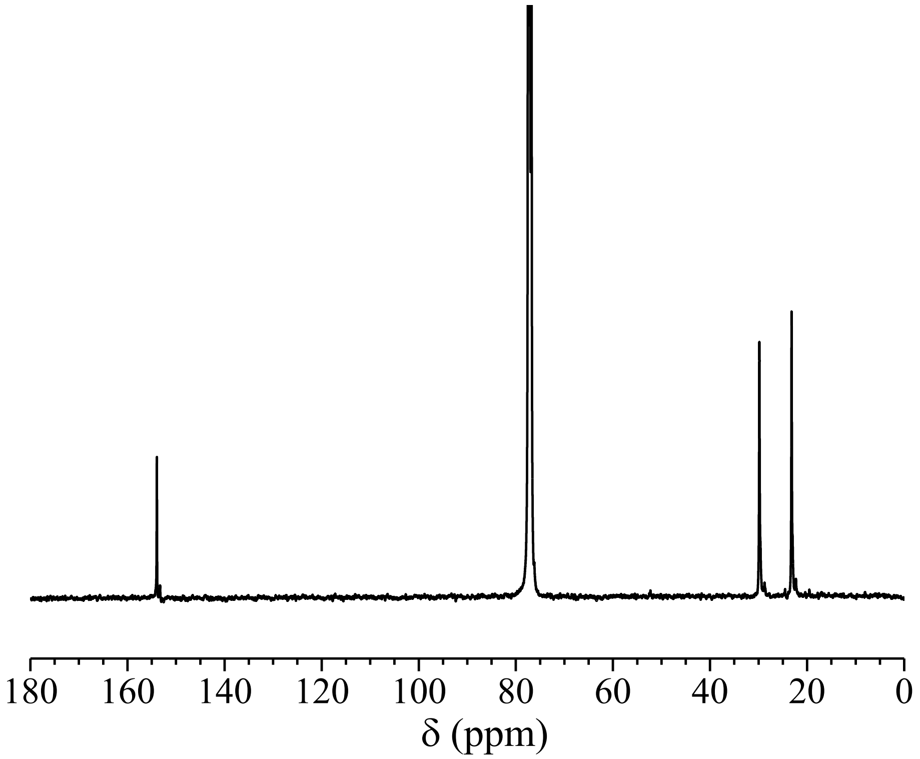

Figure S4. ^{13}C NMR spectrum of (*S*)-PCHC (solvent: δ = 77.22 ppm).

3.2 X-ray diffraction of as-polymerized samples and annealed samples

As-polymerized samples of the enantiopure isotactic (*R*)-PCHC and (*S*)-PCHC are amorphous, as shown by the X-ray powder diffraction profiles of Figure S5.

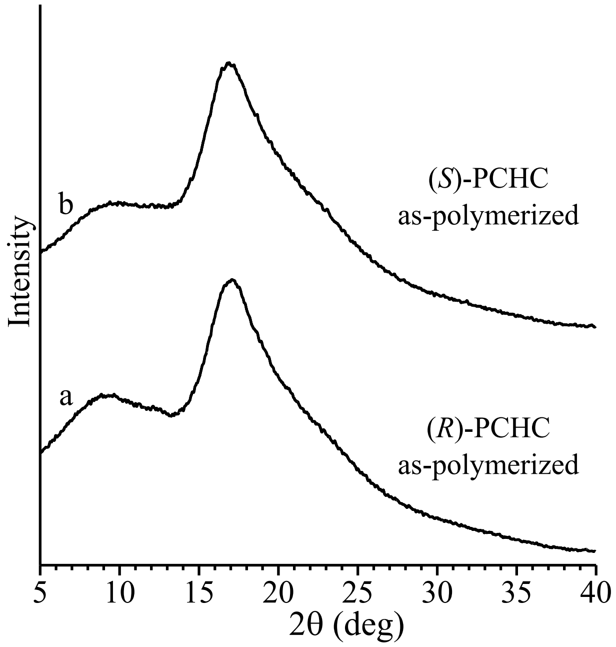

Figure S5. X-ray powder diffraction profiles of as-polymerized samples of the enantiopure isotactic (*R*)-PCHC (a) and (*S*)-PCHC (b).

Both (*R*)-PCHC and (*S*)-PCHC crystallize after annealing of the as-polymerized samples at temperature higher than 180 °C. The X-ray powder diffraction profiles of the crystalline samples annealed at 180 °C for 4 hours are reported in Figure 6. They show four main sharp and well resolved reflections, centered at about 2θ ≈ 12.1°, 17.2°, 18.8° and 20.3° and other minor reflections of lower intensities. Identical diffraction profiles are shown by the two enantiomeric samples (*R*)-PCHC and (*S*)-PCHC (Figure S6).

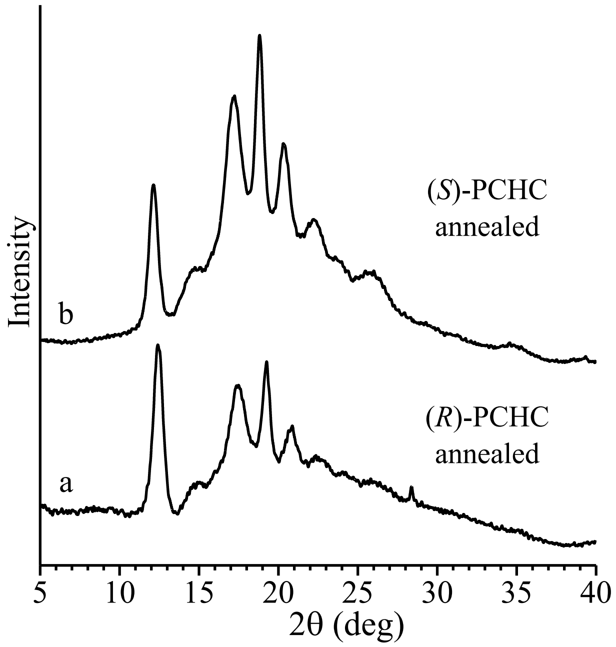

Figure S6. X-ray powder diffraction profiles of samples of (*R*)-PCHC (a) and (*S*)-PCHC (b) annealed at 180 °C for 4 hours.

3.3 X-ray fiber diffraction

The bi-dimensional X-ray diffraction pattern of crystalline oriented fibers of (*S*)-PCHC, prepared by stretching the amorphous cast film at 180 °C is reported in Figure S7. The pattern presents sharp and strong reflections on the equator at 2θ = 12.2°, 18.7° and 20.3° and a strong reflection at 2θ = 17.3° on the first layer line. These diffraction spots are at the same Bragg angles as those of the four strong reflections observed in the powder diffraction profiles of Figure S6. This indicates that the fiber is crystallized in the same crystalline form as the powder annealed cast sample. Moreover, the presence of the strong reflection at 2θ = 17.3° on a well-defined first layer line indicates an ordered conformation of the chains and a chain periodicity of c = 7.4 Å. The enantiomeric sample (*R*)-PCHC shows similar X-ray fiber diffraction pattern.

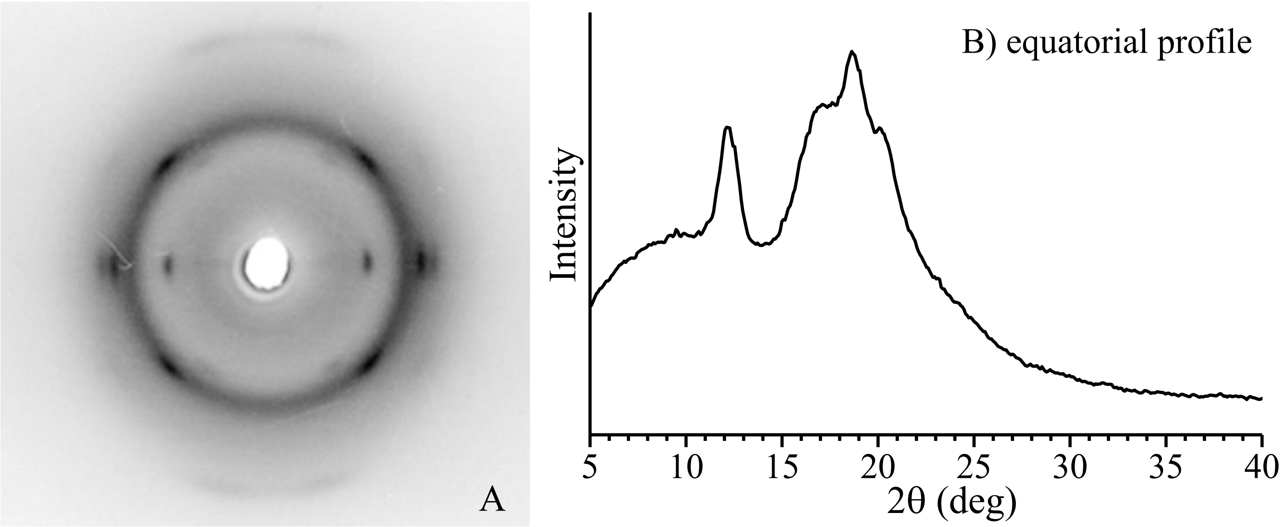

Figure S7. X-ray fiber diffraction pattern (A) and corresponding profile read along the equatorial layer line (B) of fibers of (*S*)-PCHC prepared by stretching cast film of (*S*)-PCHC at 180 °C at 500% deformation.

3.4. Morphology

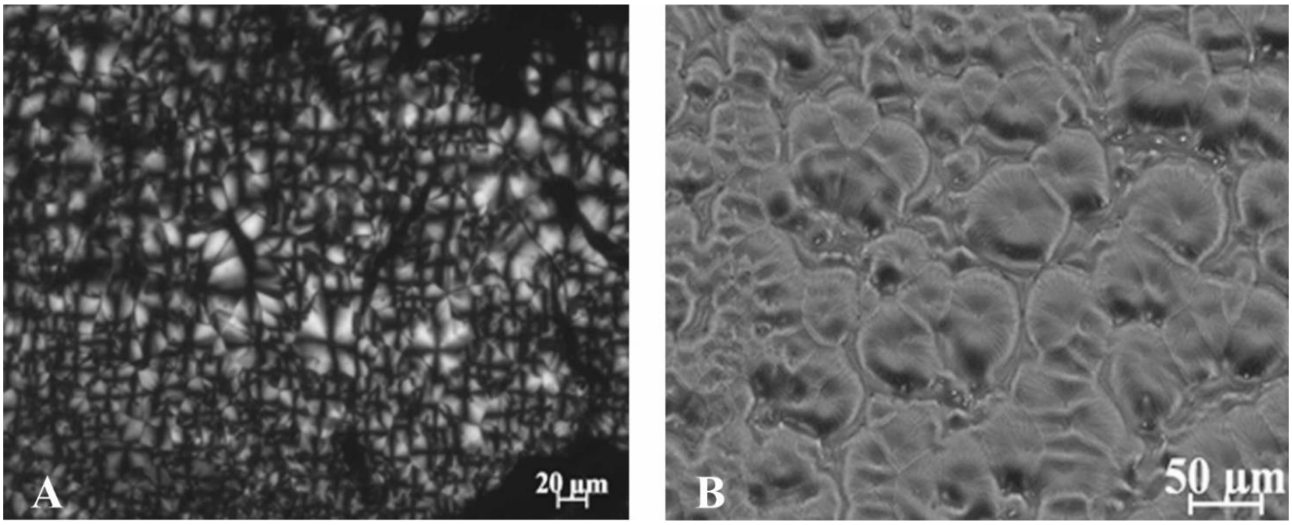

Figure S8. POM images in bright field (A) and phase contrast (B) modes of samples of (*S*)-PCHC crystallized by cooling from the melt at cooling rate of 10 °C/min.

4. Conformational Analysis.

The calculations of the conformational energy have been performed using methods of the density functional theory (DFT) on a portion of the chain of the *S*-enantiomer shown in Figure S9, specifically on the model *trans*-(*S*,*S*)-(2-methoxycarbonyloxycyclohexyl) methyl carbonate, whose conformation is defined by the five torsion angles θ_1, θ_2, θ_3, θ_3', θ_2'. The two enantiopure polymers, (*S*)-PCHC and (*R*)-PCHC, are characterized by a regular succession of *S,S* and *R,R* stereocenters, respectively, which are located at the two vicinal chiral methine carbon atoms belonging to the cyclohexyl ring (Figure S9 for (*S*)-PCHC). Therefore, the portion of chain in Figure S9 is representative of the constitution and conformation of the enantiopure polymer (*S*)-PCHC. Identical results have been obtained for the *R*-enantiomer.

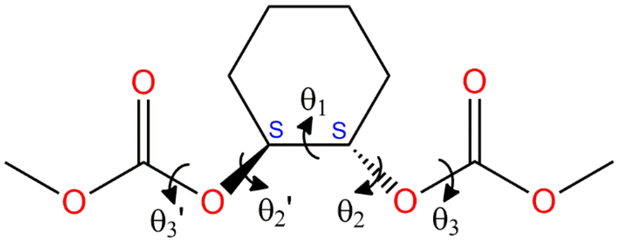

Figure S9. Portion of the chain of (*S)*-PCHC and definition of the torsion angles θ_1, θ_2, θ_2' θ_3, θ_3'. Hydrogen atoms are not shown.

Low-energy conformations were built up by fixing the cyclohexyl rings in the chair conformation and assuming the torsion angle θ_1, defined by the four O-(*S*)CH-(*S*)CH-O atoms with (*S*)C and (*S*)C belonging to cyclohexyl ring, in the *gauche* conformation, $\theta_1 \approx 60°$ (G^+), as any slight variation from G^+ would result in drastic deviation of both $O\hat{C}H$ bond angles from the tetrahedral value. Moreover, the torsion angles θ_3 and θ_3' were initially assumed at 180° (T), a common minimum energy value for dihedral angle involving ester and carbonate bonds.

The map of conformational energy for (*S*)-PCHC as a function of torsion angles θ_2 and θ_2' with θ_1, θ_3 and θ_3' freely optimized is reported in Figure S10. The map presents three non-equivalent energy minima at values of torsion angles reported in Table S1. The absolute minimum **a** is at θ_2 = -150° (A^-) and θ_2' = -150° (A^-) in a very large region of low energy which extends from 180° to -90° for both θ_2 and θ_2' where the relative energy is never higher than 1 kJ/mol with respect to absolute minimum **a**. The two relative minima **b** at θ_2 = 60° (G^+) and θ_2' = -90° (G^-) and **b'** at θ_2 = -90° (G^-) and θ_2' = 60° (G^+) are symmetrically equivalent and are 2.4 kJ/mol higher in energy with respect to the absolute minimum **a**. Finally, the third relative minimum **c** at θ_2 = 60° (G^+) and θ_2' = 60° (G^+) is a much higher energy of 14.8 kJ/mol. For all the minima, the value of the freely optimized torsion angles θ_3 and θ_3' never deviated significantly from 180° (T) and the torsion angle θ_1 remains close to

60°. Identical energy minima have been obtained in the map of the enantiomer (*R*)-PCHC with enantiomorphous conformations with opposite sign of the values of the torsion angles (minimum **a** at θ_2 = +150° (A^+) and θ_2' = +150° (A^+), minimum **b** at θ_2 = -60° (G^-) and θ_2' = +90° (G^+) and **b'** at θ_2 = +90° (G^+) and θ_2' = -60° (G^-), minimum **c** at θ_2 = -60° (G^-) and θ_2' = -60° (G^-).

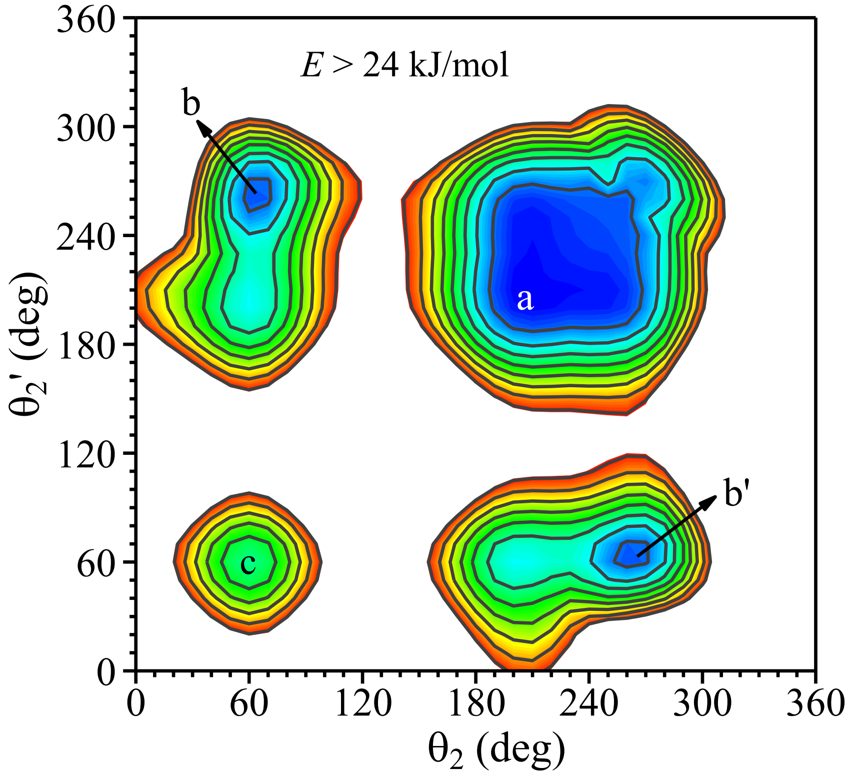

Figure S10. Maps of the conformational energy of (*S*)-PCHC as a function of the torsion angles θ_2 and θ_2' with torsion angles θ_1, θ_3 and θ_3' freely optimized (see Figure S8). The levels of energy are reported every 3 kJ/mol. The energy minima are indicated with letters. The minimum **a** is the absolute energy minimum placed at $E = 0$ kJ/mol and the energy values are scaled with respect to the absolute minimum. The colors indicate the scale of energy that decreases from red to blue.

Table S1. Values of torsion angles θ_1, θ_2, θ_3, θ_3', θ_2' and energy of the minima observed in the map of (*S*)-PCHC Fig. S10 and corresponding minima in the equivalent map of the enantiomer (*R*)-PCHC.

Energy minima	enantiomer	θ_3' (deg)	θ_2' (deg)	θ_1 (deg)	θ_2 (deg)	θ_3 (deg)	conformation	Energy (kJ/mol)
**a**	(*S*)-PCHC	180	-150	63	-150	180	$TA^-G^+A^-T$	0.0
	(*R*)-PCHC	180	+150	-63	+150	180	$TA^+G^-A^+T$	0.0
**b**	(*S*)-PCHC	180	60	60	-90	180	$TG^+G^+G^-T$	2.4
	(*R*)-PCHC	180	-60	-60	+90	180	$TG^-G^-G^+T$	2.4
**b'**	(*S*)-PCHC	180	-90	60	60	180	$TG^-G^+G^+T$	2.4
	(*R*)-PCHC	180	+90	-60	-60	180	$TG^+G^-G^-T$	2.4
**c**	(*S*)-PCHC	180	60	47	60	180	$TG^+G^+G^+T$	14.8
	(*R*)-PCHC	180	-60	-47	-60	180	$TG^-G^-G^-T$	14.8

Only the deepest energy minimum **a** at $\theta_2 = \theta_2' = -150°$ (A^-) for (*S*)-PCHC (and $\theta_2 = \theta_2' = +150°$ (A^+) for (*R*)-PCHC), matches the experimental periodicity of 7.4 Å and a repetition after two monomers. This conformation of lowest energy corresponds to a two-fold 2/1 helical conformation and a sequence of torsion angles $(\theta_3', \theta_2', \theta_1, \theta_2, \theta_3)_n = (180°, -150°, 63°, -150°, 180°)_n = (TA^-G^+A^-T)_n$ for (*S*)-PCHC, and $(\theta_3', \theta_2', \theta_1, \theta_2, \theta_3)_n = (180°, +150°, -63°, +150°, 180°)_n = (TA^+G^-A^+T)_n$ for (*R*)-PCHC.

The conformations corresponding to the relative minima **b** and **b**' are not feasible because the sequence of two consecutive bonds with opposite *gauche* torsion angles G^+G^- gives folded conformations.

The 2/1 helical conformation has been then optimized by minimization of the energy while changing the five torsion angles and bond angles and lengths. Minimization has been executed by DFT calculations with Periodic Boundary Condition (PBC) and the restriction of repetition after two monomers and a periodicity corresponding to the experimental chain axis of 7.4 Å. The optimization gives a two-fold helical conformation with 2/1 symmetry with a sequence of torsion angles $(\theta_3', \theta_2', \theta_1, \theta_2, \theta_3)_n = (167.6°, -94.7°, 68.0°, -141.2°, -176.4°)_n$ for (*S*)-PCHC and the enantiomorphous conformation $(\theta_3', \theta_2', \theta_1, \theta_2, \theta_3)_n = (-167.6°, 94.7°, -68.0°, 141.2°, 176.4°)_n$ for the enantiomer (*R*)-PCHC, and a periodicity of 7.36 Å, which perfectly matches the measured value of the *c* axis.

Remarkably, this periodicity of 7.36 Å is achieved spontaneously for the minimum energy conformation without cost of energy. Even though the torsion angle θ_3 and θ_2 (and θ_3' and θ_2') slightly deviate from the perfect T and A conformations, respectively, we still define this two-fold helical conformation with s(2/1) symmetry by the sequence of torsion angles $(TA^-G^+A^-T)_n$ for the (*S*)-PCHC chain and $(TA^+G^-A^+T)_n$ for the (*R*)-PCHC chain.

A model of the 2/1 helical conformation of (*S*)-PCHC chain is shown in Figure 3B of the main text.

5. Simulated X-ray fiber diffraction

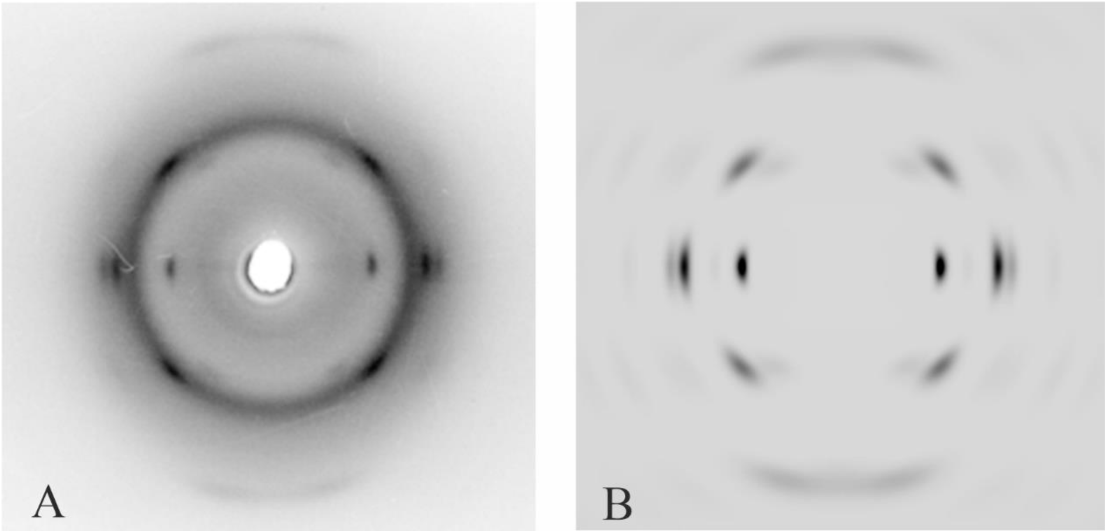

Figure S11. Comparison between experimental X-ray fiber diffraction pattern of stretched and annealed fibers of (*S*)-PCHC (A) and calculated X-ray fiber diffraction pattern for the model of the crystal structure of (*S*)-PCHC of Figure 3C.C'.

6. Crystal structure of the enantiopure (*S*)-PCHC

Table S2. Fractional coordinates of the asymmetric unit in the model of the crystal structure of (*S*)-PCHC of Figure 3C,C' in the orthorhombic unit cell with axes a = 11.55 Å, b = 9.42 Å and c = 7.36 Å, according to the space group $P2_12_12_1$.

Atom	Type atom	*x*/*a*	*y*/*b*	*z*/*c*
1	C	0.6275	0.0444	0.1100
2	C	0.5015	0.0666	0.1688
3	C	0.4179	0.0082	0.0249
4	C	0.4403	0.0700	0.8345
5	C	0.5661	0.0490	0.7766
6	C	0.6498	0.1088	0.9201
7	O	0.3008	0.0464	0.0749
8	C	0.2392	-0.0526	0.1655
9	O	0.2692	-0.1723	0.1997
10	O	0.1365	0.0086	0.2073
11	H	0.4838	0.1799	0.1859
12	H	0.4833	0.0157	0.3016
13	H	0.6469	-0.0692	0.1058
14	H	0.6861	0.0905	0.2129
15	H	0.4234	-0.1073	0.0185
16	H	0.4157	0.1818	0.8332
17	H	0.5794	0.0982	0.6412
18	H	0.5817	-0.0647	0.7590
19	H	0.6391	0.2241	0.9281
20	H	0.7396	0.0896	0.8764

7. Stereocomplex (*R*/*S*)-PCHC

A sample of 1:1 blend of (*R*)-PCHC and (*S*)-PCHC was prepared by dissolving equal amounts of the two enantiopure polymers in CH_2Cl_2 solution and successive casting at room temperature. The blend has been isothermally crystallized from the melt at 225 °C for 2h.

7.1 X-ray diffraction of the stereocomplex (*R*/*S*)-PCHC.

The diffraction profile of the melt-crystallized blend is reported in Figure S12. The diffraction profile of Figure S12 is similar to that of the stereocomplex reported by Lu et al. in the ref. [12]. However, the diffraction profile of Figure S12 shows, besides the reflections of the stereocomplex, weak reflections of the enantiopure polymers (profile b of Figure 2A and Figure S6). This indicates that in our 1:1 blend the crystallization of the stereocomplex is not complete and part of the blend crystallizes giving small amount of separate crystals of the enantiopure polymers (*S*)-PCHC and (*R*)-PCHC.

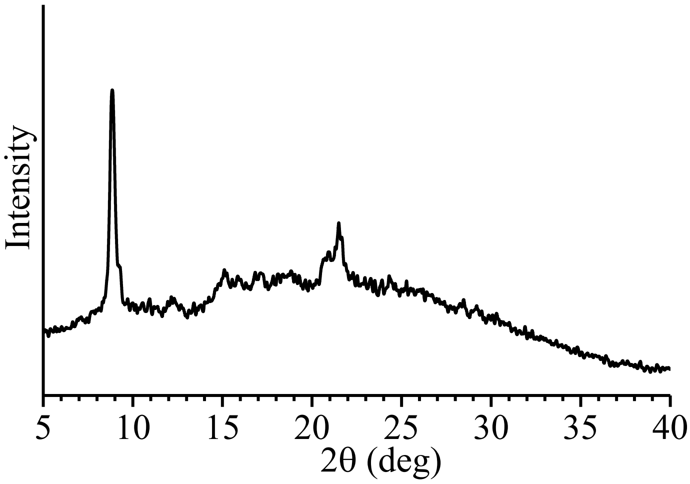

Figure S12. X-ray powder diffraction profile of the stereocomplex (*R*/*S*)-PCHC crystallized from solution of the 1:1 blend of the enantiopure polymers (*S*)-PCHC and (*R*)-PCHC after isothermal crystallization from the melt at 225 °C for 2 h.

7.2 FTIR spectra of enantiopure (*S*)-PCHC and of the stereocomplex (*R*/*S*)-PCHC.

The FTIR spectra of samples of the enantiopure (*S*)-PCHC and of the stereocomplex (*R*/*S*)-PCHC are compared in Figure S13. It is apparent that the band of the carbonate group at 1749 cm^{-1} in the enantiopure (*S*)-PCHC moves to 1759 cm^{-1} in the spectrum of the stereocomplex with a wavenumber shift of 10 cm^{-1}. This shift to higher wavenumbers indicates a strengthening of the C=O double bond as a result of establishment of strong non-covalent interactions involving the carbonate groups. Since the structure of the enantiopure (*S*)-PCHC is already characterized by the presence of H---O=C attractive interactions at short distance of 2.38 Å between oxygen atoms of carbonyl groups and the hydrogen atoms of the methylene groups of the cyclohexyl rings (Figure 3C,C'), the shift of the

carbonyl band in the stereocomplex indicates the presence, besides the H---O=C attractive interactions, of additional interactions, such as the C=O---C=O intimate dipole interactions between carbonyl groups of chains of opposite chirality (as shown in the model of the crystal structure of Figure 6). We hypothesize that this directional inductive bonding strengthens the double bond and suppresses the conjugation effect.

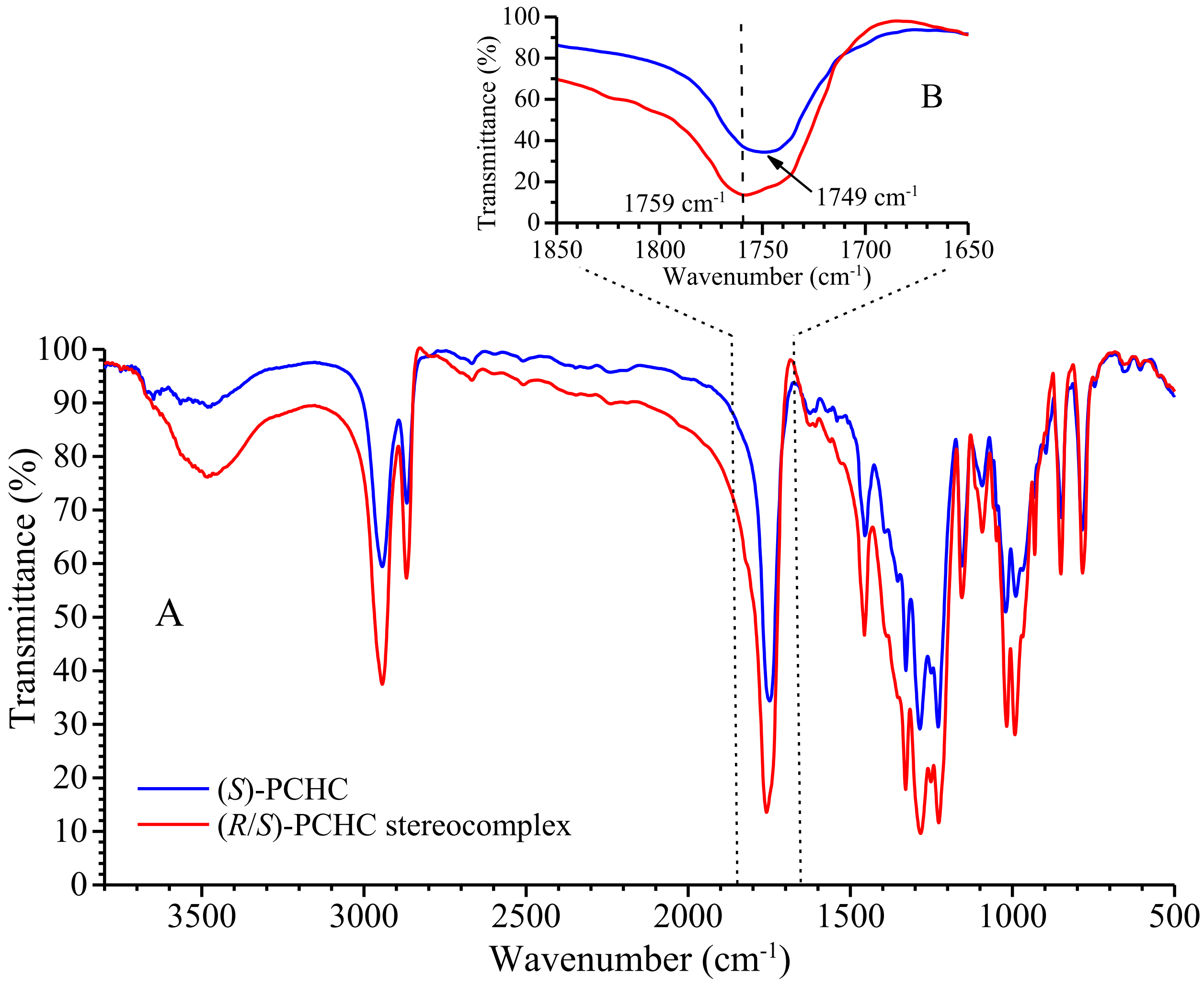

Figure S13. FTIR spectra of the samples of the enantiopure (*S*)-PCHC (blue) and of the stereocomplex (*R*/*S*)-PCHC (red) (A), and enlarged region between 1650-1850 cm^{-1} of the band of the carbonyl groups (B). The stereocomplex has been crystallized from solution of the 1:1 blend of the enantiopure polymers (*S*)-PCHC and (*R*)-PCHC after isothermal crystallization from the melt at 225 °C for 2 h.

7.3 Analysis of non-covalent interactions in the crystals structures of enantiopure (*S*)-PCHC and of the stereocomplex (*R*/*S*)-PCHC.

We have analyzed the chain-chain interactions present in the proposed models of the crystal structures of the enantiopure polymer (*S*)-PCHC (Figure 3C,C') and of the stereocomplex (*R*/*S*)-PCHC (Figure 6). The analysis of the non-covalent interactions (NCIs) has been performed using the software NCIPLOT[S14] and visualized by VMD.[S15] Representative pairs of polymeric chains were

selected based on the proposed models of the crystal structures (Figures 3 and 6). This choice was made in order to preserve, as accurately as possible, the intermolecular interactions occurring in the crystals, as dictated by the crystal symmetry. The results are reported in Figure S14. Significant attractive interactions are clearly present in both the structures of the enantiopure (*S*)-PCHC (Figure S14A) and of the stereocomplex (*R*/*S*)-PCHC (Figure S14B). The interactions can be considered increasingly stronger and more attractive when moving from green to blue. In particular, it can be observed that in the enantiopure (*S*)-PCHC a small turquoise disc appears between the C=O and H–C groups (Figure S14A), whereas a large bluish region is visible between two carbonyl C=O groups in the stereocomplex (Figure S14B). This confirms the presence of H---O=C attractive interactions in both the structures of the enantiopure polymer (*S*)-PCHC and of the stereocomplex, and additional C=O---C=O intimate dipole interactions in the structure of the stereocomplex (*R*/*S*)-PCHC.

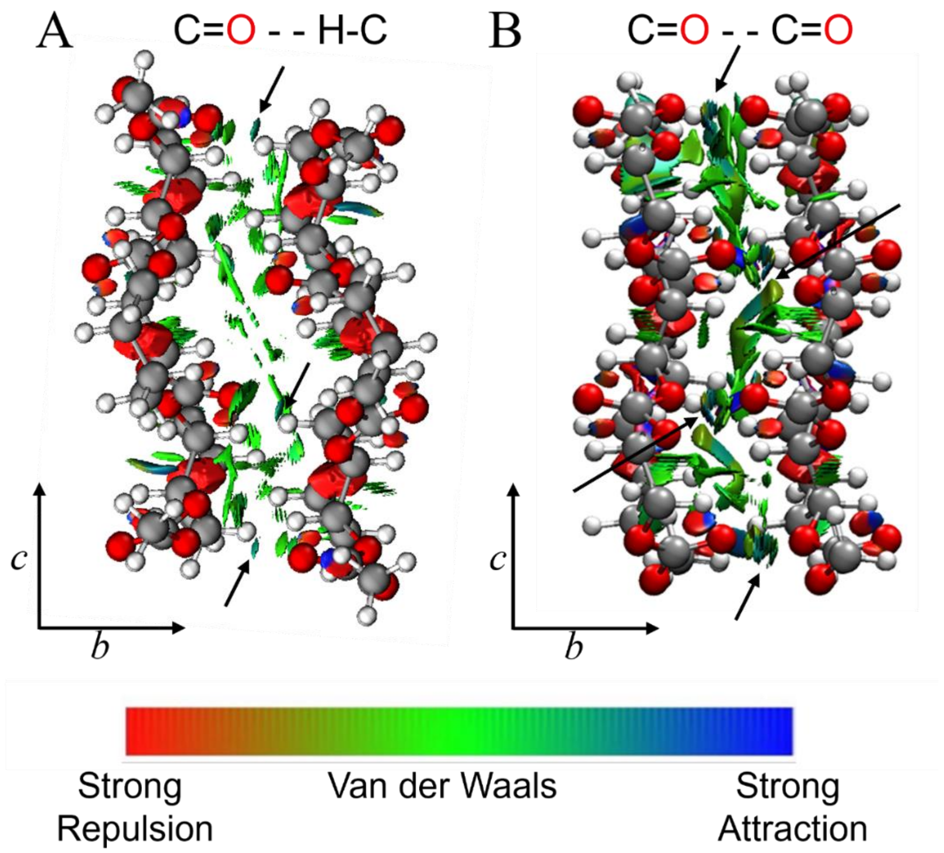

Figure S14. Gradient isosurfaces in the crystal structures of the enantiopure (*S*)-PCHC (A) and of the stereocomplex (*R*/*S*)-PCHC (B) showing non-covalent interactions. Black arrows indicate the slightly attractive H---O=C interactions (green-blue) in A and the attractive dipole C=O---C=O interactions (blue) in B. NCI surface corresponds to density reduced gradient s = 0.35 au and a color scale of density from -3 to +3 au for Self-Consistent Field (SCF) densities.

8. References.

[S1] W. C. Ellis, Y. Jung, M. Mulzer, R. Di Girolamo, E. B. Lobkovsky, G. W. Coates, *Chem. Sci.*, **2014**, *5*, 4004–4011.

[S2] Gaussian 09, Revision D.01, M. J. Frisch, G.W. Trucks, H. B. Schlegel, G. E. Scuseria, M. A. Robb, J. R. Cheeseman, G. Scalmani, V. Barone, B. Mennucci, G. A. Petersson, H. Nakatsuji, M. Caricato, X. Li, H.P. Hratchian, A. V. Izmaylov, J. Bloino, G. Zheng, J. L. Sonnenberg, M. Hada, M. Ehara, K. Toyota, R. Fukuda, J. Hasegawa, M. Ishida, T. Nakajima, Y. Honda, O. Kitao, H. Nakai, T. Vreven, J.-A., Jr. Montgomery, J. E. Peralta, F. Ogliaro, M. Bearpark, J. J. Heyd, E. Brothers, K. N. Kudin, V. N. Staroverov, R. Kobayashi, J. Normand, K. Raghavachari, A. Rendell, J. C. Burant, S. S. Iyengar, J. Tomasi, M. Cossi, N. Rega, N. J. Millam, M. Klene, J. E. Knox, J. B. Cross, V. Bakken, C. Adamo, J. Jaramillo, R. Gomperts, R. E. Stratmann, O. Yazyev, A. J. Austin, R. Cammi, C. Pomelli, J. W. Ochterski, R. L. Martin, K. Morokuma, V. G. Zakrzewski, G. A. Voth, P. Salvador, J. J. Dannenberg, S. Dapprich, A. D. Daniels, Ö. Farkas, J. B. Foresman, J. V. Ortiz, J. Cioslowski, D. J. Fox, Gaussian, Inc., Wallingford CT, **2009**.

[S3] J. P. Perdew, K. Burke, M. Ernzerhof, *Phys. Rev. Lett.* **1996**, *77*, 3865-3868.

[S4] J. P. Perdew, K. Burke, M. Ernzerhof, *Phys. Rev. Lett.* **1997**, *78*, 1396.

[S5] S. Grimme, *J. Comput. Chem*. **2004**, *25*, 1463−1473

[S6] S. Grimme, J. Antony, S. Ehrlich, H. A. Krieg, *J. Chem. Phys.* **2004** *132*, 154104.

[S7] G. A. Petersson, A. Bennett, T. G. Tensfeldt, M. A. Al-Laham, W. A. Shirley, J. Mantzaris, *J. Chem. Phys*. **1988**, *89*, 2193-2218.

[S8] G. A. Petersson, M. A. Al-Laham, *J. Chem. Phys.*, **1991**, *94*, 6081-6090.

[S9] B. Delley, *J. Chem. Phys*. **1990**, *92*, 508-517.

[S10] B. Delley, *J. Chem. Phys*. **2000**, *113*, 7756-7764.

[S11] H. M. Rietveld, *J. Appl. Cryst*. **1969**, *2*, 65-71.

[S12] R. A. Young, The Rietveld Method, IUCr Monographies of Crystallography, 5, Oxford University Press, Oxford **1993**.

[S13] International Table for X-ray crystallography, Vol. B: Reciprocal Space, Shmueli U. ed., Springer, Dordrecht, **2008**.

[S14] R. A. Boto, F. Peccati, R. Laplaza, C. Quan, A. Carbone, J.-P. Piquemal, Y. Maday, J. Contreras-García, *J. Chem. Theory Comput*. **2020**, *16*, 4150–4158.

[S15] W. Humphrey, A. Dalke, K. Schulten, *J. Mol. Graph.* **1996**, *14*, 33–38.
